# THE IMPACT OF THE TEACHING NURSING HOME ON THE NURSING HOME WORKFORCE

**DOI:** 10.1093/geroni/igae098.3243

**Published:** 2024-12-31

**Authors:** Howard Degenholtz, Kenna Campbell, Anny Treat

**Affiliations:** University of Pittsburgh, Pittsburgh, Pennsylvania, United States; University of Pittsburgh, Pittsburgh, Pennsylvania, United States; Sebastian Mann Associates, Pittsburgh, Pennsylvania, United States

## Abstract

The Teaching Nursing Home (TNH) is designed to improve the long-term care workforce and improve quality of care in nursing homes. The initiative has multiple components: enhanced clinical rotations for nursing students with partner schools of nursing, implementation of the Institute for Healthcare Improvement Age-Friendly Health System “4M” quality improvement model, and an online learning network. CMS Payroll Based Journal (PBJ) data were analyzed using a propensity weighted difference in difference model to determine if there was an effect on attrition or staffing levels. Staff satisfaction and burnout surveys were conducted at participating nursing homes in the winter of 2022 using a combination of web-based and in-person paper administration. Follow-up surveys were collected in 2023. Response rates ranged from 14% to 54%. A total of 190 surveys were available at baseline, and 183 at follow-up. All surveys were anonymous; cross-sectional analyses were conducted to identify trends. The findings for burnout were mixed. Emotional exhaustion decreased, but depersonalization increased and personal accomplishment was unchanged. Overall job satisfaction was unchanged but the management and leadership component of fob satisfaction declined. Attrition among registered nurses, licensed practical nurses and certified nurse assistants was lower than among matched facilities in the same market areas. After the first year of implementation, the TNH program is showing mixed benefits in terms of burnout and job satisfaction, but some benefit in terms of reducing turnover. As the Age-Friendly Nursing Home program is disseminated across the US, it is important to track the impact on the workforce.

